# The Cincinnati Polyclinic

**Published:** 1889-05

**Authors:** 


SCHOOLS FOR POST-GRADUATE INSTRUCTION are being rapidly organized in all the larger cities. One of the latest is the Cincinnati Polyclinic, the announcement of which has just come to hand. It contains the names of many of the well-known teachers there, and bids fair to be a prosperous school. We notice that the teachers have been selected from all the medical colleges of that city, and this seems to us a method to be commended. It will certainly tend to unite the profession closer than before. We wish this method could be followed in other places. We feel sure that the Cincinnati Polyclinic will take rank with any school of the kind in this country.

